# Comparison of Antioxidant Properties and Metabolite Profiling of *Acer pseudoplatanus* Leaves of Different Colors

**DOI:** 10.3390/antiox12010065

**Published:** 2022-12-28

**Authors:** Ming Zhang, Jeehwan Choe, Ting Bu, Shuilin Liu, Sooah Kim

**Affiliations:** 1 Department of Environment Science and Biotechnology, Jeonju University, Jeonju 55069, Republic of Korea; 2 Department of Livestock, Korea National College of Agriculture and Fisheries, Jeonju 54874, Republic of Korea; 3 College of Horticulture, Hebei Agricultural University, Baoding 071000, China

**Keywords:** antioxidant, different color, maple leaves, metabolites, polyphenols

## Abstract

*Acer pseudoplatanus* (maple) is a widely grown ornamental plant. In addition to its ornamental and ecological value, it also has potentially high economic value. It is a rich source of polyphenols and exhibits antioxidant activity. However, the relationship between polyphenol content and antioxidant activity in maple leaves of different colors (green, yellow, and red) has not yet been investigated. In this study, the total polyphenol (TP), total flavonoid (TFlav), tannin (TET), chlorophyll a and b (Chl a and b), total anthocyanin (TAN), and total carotene (TAC) contents in maple leaves of different colors were evaluated. Their antioxidant activities were determined based on the inhibition of lipid oxidation, DPPH scavenging, ferric ion-reducing antioxidant power, and iron-chelating abilities. The concentrations of TP, TET, TFlav, TAN, and TAC in red maple leaves were higher than those in green and yellow maple leaves. In addition, red maple leaves showed a higher antioxidant effect than the leaves of the other two colors. We observed that antioxidant activity was positively correlated with TP, TFlav, and TAN and negatively correlated with Chl a and b. Finally, we analyzed the metabolites of the different colored (i.e., green, yellow, and red) maple leaves using gas chromatography/mass spectrometry (GC/MS) and found that the metabolite profile significantly varied between the different colors. These results suggest that red leaves are a good source of polyphenols and antioxidants and have potential use in the development of functional foods and medicinal applications.

## 1. Introduction

Oxidative stress disrupts the balance between reactive oxygen species (ROS), free radicals, and antioxidant defenses [1]. It can play a crucial role in the development of various diseases, such as cancer [2], malaria [3], arteriosclerosis [4], rheumatoid arthritis [5], and neurodegenerative diseases [6], and the aging process [7]. Antioxidants are synthesized in the human body or taken up from the environment through diet [8]. Although synthetic antioxidants are widely used in the food, medicine, cosmetics, and other fields, there are safety issues associated with these agents. Previous research has indicated that long-term intake of synthetic antioxidants is associated with certain teratogenic and carcinogenic risks [9].

Bioactive compounds, including polyphenols, as a nutritional component, are usually presented small amounts in food, and have been reported to have various health effects, such as antioxidation, bacteriostasis, anti-inflammation, and immunity enhancement. Therefore, bioactive substances have been widely used in foods, pharmaceuticals, and cosmetics [10,11,12,13]. Polyphenols play an important role as antioxidants. Although human diets such as fruits [14], vegetables [15], tea [16], and wine [17] are rich in polyphenols, it is also necessary to find a natural, safe, and economic antioxidant in industrial production for sustainability [18]. Previous studies have found that olive rape [19], durum wheat bran [20], *Fraxinus ornus* bark [21], and potato peel waste [22] are good sources of natural polyphenols. In particular, maple leaves are inexpensive, easy to obtain, and have significant value for the rational use of resources and sustainable development.

Anthocyanins, which are flavonoids, play an important role in physiological and biochemical processes in plants, such as leaf color change. When leaves are subjected to biotic and abiotic stresses, they are synthesized and accumulate in plant vacuoles [23,24]. In autumn, under the influence of photoperiod and low temperature, maple leaves begin to senesce, chlorophyll content begins to decline, and anthocyanin content begins to rise; therefore, maple leaves gradually change color from green to red [25]. Previous studies have shown that reactive oxygen species (ROS) levels increase and antioxidant capacity decreases during leaf senescence [26,27].

Previous studies on the antioxidant capacity of maple products included analyses of maple syrup [28], maple sap [29], bark extracts [30], and leaf extracts [31]. However, to the best of our knowledge, no study has compared the antioxidant capacity and metabolite profiles of maple leaves of different colors simultaneously.

Therefore, the present study aimed to investigate the antioxidant potential of different colored maple leaves and compare the content of total polyphenols, flavonoids, tannins, chlorophyll a and b, and carotenoids in green, yellow, and red leaves. The antioxidant properties of green, yellow, and red leaves were determined using four different methods: thiobarbituric acid reactive substances (TBARS), 2,2-diphenyl-1-picrylhydrazyl radical (DPPH), metal chelation, and ferric ion-reducing antioxidant power (FRAP). Finally, to investigate the metabolic pathways associated with the colors, metabolite profiling of green, yellow, and red leaves was conducted using gas chromatography/mass spectrometry (GC/MS).

## 2. Materials and Methods

### 2.1. Maple Leaves

**Figure 1:**
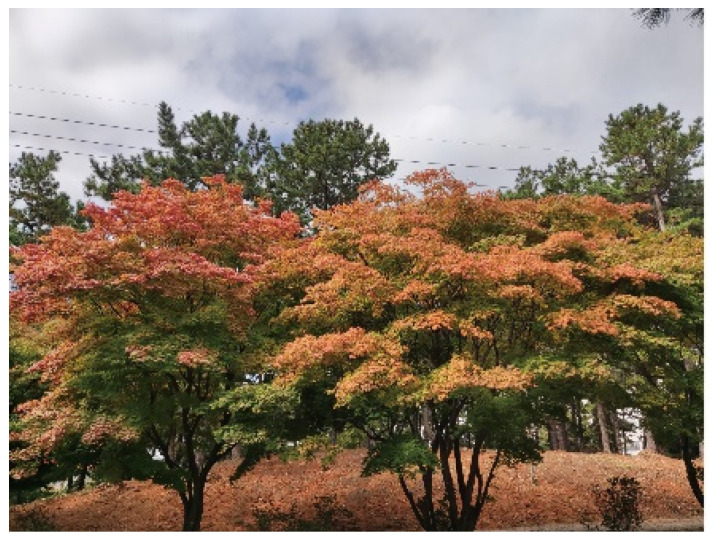
Maple leaves of different colors in Jeonju University.

Maple leaves of different colors (green, yellow, and red) were collected from Jeonju University (Jeonju, Jeonbuk, Republic of Korea) on 6 November 2020 (Figure 1). The leaves collected from multiple trees were divided into three groups according to their color and then dried at 40 °C to constant weight. The dried leaves were ground using a mortar and pestle and stored at −60 °C until use.

### 2.2. Polyphenol Content

One gram of sample was extracted with 20 mL of 50% aqueous methanol (JT Baker, Radnor, PA, USA) for 3 h at 25 °C, with shaking at 200 rpm. The sample was centrifuged at 195× *g* for 15 min at 25 °C (1580R, Labogene, Bjarkesvej, Lillerød, Denmark), then the supernatant was collected [1,32,33].

#### 2.2.1. Total Polyphenol (TP)

The Folin–Ciocalteu method was used to determine the TP content [32]. Briefly, 16 μL of the extract and 60 μL of the Folin–Ciocalteu reagent (Sigma-Aldrich, St. Louis, MO, USA. Unless otherwise stated, all reagents are purchased from Sigma-Aldrich) were mixed for 5 min at 25 °C. Sixty microliter of 60 g/L Na_2_CO_3_ were added and incubated for 90 min at 25 °C. The absorbance of the mixture was measured at 725 nm (Multiskan SkyHigh, Thermo Fisher Scientific, Waltham, MA, USA). TP was expressed as milligrams of gallic acid equivalents per gram of dry weight (mg GAE/g DW).

#### 2.2.2. Total Extractable Tannin (TET)

The TET content was measured as previously described [18]. Briefly, to precipitate tannins, 110 mg of PVPP (Polyvinylpyrrolidon) was added to 1 mL of the extract, mixed, and centrifuged at 970× *g* for 10 min at 4 °C (1730R, Labogene). The TP content in the supernatant (corresponding to the non-precipitated phenols) was determined using the Folin–Ciocalteu method, as described in Section 2.2.1. The TET content was calculated by subtracting the non-precipitated phenols from TP.

#### 2.2.3. Total Flavonoid Content (TFlav)

The TFlav content was determined as described previously [34]. Briefly, 20 μL of the extract was mixed with 80 μL of ddH_2_O (double distilled H_2_O), 6 μL of 5% NaNO_2_ solution, and 6 μL of 10% AlCl_3_ solution for 6 min, followed by addition of 80 μL of 4% NaOH (Duksan, Ansan-si, Gyeonggi-do, Republic of Korea) solution. The mixture was incubated in the dark for 30 min at 25 °C, and the absorbance was measured at 510 nm (Multiskan SkyHigh, Thermo Fisher Scientific). The results are expressed in mg QE/g DW (QE, quercetin equivalents).

#### 2.2.4. Chlorophyll Content

The chlorophyll content was determined using a previously described method [34]. Briefly, 100 mg of leaves was mixed with 4 mL of 80% acetone (JT Baker). After rinsing several times with 80% acetone, the sample was completely transferred to a test tube and the volume was adjusted to 10 mL with acetone. The mixture was centrifuged immediately at 1763× *g* for 10 min (1580R, Labogene). The absorbance of the mixture was measured at 646.8 nm and 663.2 nm (UV-1800, Shimadzu, Kyoto, Japan). The chlorophyll concentration was calculated using the following formula:

Chl a (mg/L) = (12.25 × D_663.2_) − (2.79 × D_646.8_),

Chl b (mg/L) = (21.50 × D_646.8_) − (5.10 × D_663.2_),

Chl a (mg/g) = [Chl a (mg/L) × 10 mL]/100 mg,

Chl b (mg/g) = [Chl b (mg/L) × 10 mL]/100 mg

#### 2.2.5. Anthocyanin Content

Anthocyanin content was measured in the dark, as described by Al-Farsi et al. [32,35]. A pH 1.0 buffer was prepared by mixing 14.9 mg/mL of KCl and 0.2 mol/L of HCl (Duksan) at a ratio of 25:67 and a pH 4.5 buffer of 1.64 mg/mL of sodium acetate.

For extraction, the leaves (0.25 g) were homogenized in 20 mL of distilled water for 1 min and sonicated for 15 min. One mililiter of the supernatant was transferred to a 25 mL volumetric flask after centrifugation at 440× *g* for 10 min (1580R, Labogene) and adjusted to the final volume with buffer (pH 1.0). Another 1 mL of the supernatant was transferred to a 25 mL volumetric flask and adjusted to the final volume with pH 4.5 buffer. The absorbance values of these mixtures were measured at 510 and 700 nm (UV-1800, Shimadzu). The absorbance was calculated as follows:

Ab = (Ab_510nm_ − Ab_700nm_) pH1.0 − (Ab_510nm_ − Ab_700nm_) pH4.5

The total anthocyanin content was calculated using the following equation and expressed as cyanidin 3-glucoside equivalents:

Total anthocyanin content (mg/100 g) was calculated as Ab/eL × MW × D × V/G × 100;

where e = 26,900 (molar absorptivity of cyanidin 3-glucoside), L (cell path length) = 1 mL, MW (molecular weight of anthocyanins) = 449.2, D (dilution factor) = 25, V = 25 mL, and G = 250 mg.

#### 2.2.6. Total Carotenoid Content

Total carotenoid content was measured as previously described [32]. The sample (2 g) was extracted multiple times with an acetone/ethanol (1:1) mixture containing 200 mg/L butylated hydroxytoluene until it turned colorless. After centrifugation at 440× *g* for 15 min (1580R, Labogene), the supernatant was collected. The volume of the combined supernatant was adjusted to 100 mL and the absorbance was measured at 470 nm (UV-1800, Shimadzu). Total carotenoid content was calculated using the following equation and expressed as mg per 100 g of sample weight:

Total carotenoid content (mg/g) = (Ab × V × 10^6^)/(A^1%^ × 100 G);

where Ab is the absorbance at 470 nm, V is the total volume of the extract, A^1%^ is the average extinction coefficient of carotenoids (2500 M^−1^·cm^−1^), and G is the sample weight (g).

### 2.3. In Vitro Antioxidant Test

#### 2.3.1. Evaluation of Antioxidant Activity in the Linoleic Acid Model System

The antioxidant activity of the extract was determined based on the inhibition of linoleic acid peroxidation, using the TBARS method [36,37,38].

##### Preparation and Treatment of Linoleic Acid Emulsions

Linoleic acid emulsion was prepared by mixing 300 μL of linoleic acid and Tween 20 in 50 mL of 0.2 M phosphate buffer (pH 7.2) as an emulsifier. Two milliliters of the extract (5 mg/mL DW) were mixed with 2 mL of linoleic acid emulsion in 0.2 M phosphate buffer (pH 7.0) at 50 °C for 5 days to accelerate the oxidation of lipids.

##### Thiobarbituric Acid Reactive Substances (TBARS)

Lipid oxidation was measured using the 2-thiobarbituric acid (TBA) method, as described previously, with slight modifications [39]. Briefly, 100 μL of the emulsion was mixed with 400 μL of TBA–TCA solution (20 mM TBA in 15% TCA) at 100 °C for 15 min. After cooling to 25 °C, 2 mL of chloroform (JT Baker) was added, the mixture was centrifuged at 431× *g* for 15 min at 4 °C (1730R, Labogene) and the supernatant was collected. The absorbance of the samples was measured at 532 nm using a microplate reader (Multiskan SkyHigh, Thermo Fisher Scientific). A sample containing the emulsion in TCA solution was used as a blank.

#### 2.3.2. DPPH-Free Radical Scavenging Effect

DPPH radical scavenging activity was determined according to a previously described method [40], with minor modifications. Briefly, 50 mg/mL of sample solution was diluted to different concentrations in 50% methanol. DPPH solution (100 µL) was added to 100 μL of sample solutions of different concentrations, and the mixture was incubated at 25 °C for 30 min in the dark. The absorbance of the mixture was measured at 518 nm using a microplate reader (Multiskan SkyHigh, Thermo Fisher Scientific), and the percentage antioxidant activity (AA) was calculated using the following formula:

AA% = 100 − [(Ab_sample_ − Ab_blank_ × 100)/Ab_control_];

where Ab_blank_ is the absorbance of the blank (100 μL of 50% aqueous methanol + 100 μL of sample extract solution), and Ab_control_ is the absorbance of the control (100 μL of 50% aqueous methanol + 100 μL of DPPH). The IC_50_ values of different leaves were calculated using SPSS (Statistical Package for the Social Sciences, version 25, IBM, Armonk, NY, USA).

#### 2.3.3. FRAP

FRAP assay was performed according to a previously described method, with some modifications [41]. The FRAP working reagent was prepared by mixing 25 mL of 300 mM acetate buffer (pH 3.6), 2.5 mL of 10 mM TPTZ (2,3,5-triphenyltetrazolium chloride) solution in 40 mM HCl, and 2.5 mL of 20 mM FeCl_3_·6H_2_O solution, incubated at 37 °C until further use. Twenty microliters of the extract (1.5625 mg/mL DW) was mixed with 180 μL of FRAP working reagent for 30 min in the dark. Then, the absorbances of the samples were measured at 593 nm using a microplate reader (Multiskan SkyHigh, Thermo Fisher Scientific). A standard curve of divalent iron ions was prepared using iron sulfate, and a reference experiment was conducted with ascorbic acid under the same experimental conditions. The results are expressed as ascorbic acid equivalents (AAE) per mg of dry plant material.

#### 2.3.4. Chelation Capability of Metal Ions

The free radical scavenging activity of the iron chelators was determined using a previously described method, with slight modifications [42]. 6 μL of 2 mM FeCl_2_ and 12 μL of 5 mM ferrozine solution were added to 200 μL of the extract at different concentrations (0–50 mg/mL) and then incubated at 25 °C for 10 min. The absorbance of the mixture and ethylenediaminetetraacetic acid EDTA (ethylene diamine tetraacetic acid; Duksan) control were determined at 562 nm using a microplate reader (Multiskan SkyHigh, Thermo Fisher Scientific). The ability of the extract to chelate ferrous ions was calculated using the following equation:

Chelating effect (%) = [1 − (Ab_sample_/Ab_control_)] × 100

### 2.4. Extraction and Analysis of Metabolites

Metabolites were extracted and analyzed as previously described, with slight modifications [43,44]. Briefly, 10 mg of dried sample was mixed with extraction solvents (methanol:chloroform:distilled water in a ratio of 14:4:2.85), vortexed for 30 min, and centrifuged at 24,000× *g* for 3 min at 4 °C (1730R, Labogene). Finally, 100 μL of the supernatant was vacuum dried using a vacuum concentrator (NB-503CIR, N-BIOTEK, Bucheon, Gyeonggi, Republic of Korea).

For chemical derivatization, we mixed 10 μL of methoxyamine hydrochloride in pyridine (40 mg/mL) for 90 min at 30 °C, followed by 45 μL of N-methyl-N-(trimethylsilyl) trifluoroacetamide (MSTFA) for 30 min at 30 °C. A mixture of fatty acid methyl esters (C8–C30) was used for quality control purposes. All the samples were analyzed within 24 h of derivatization.

The metabolites were analyzed using GC/MS (Shimadzu QP2010 Plus, Shimadzu) equipped with a column Rtx-5sil (30 m × 0.25 mm i.d. × 0.25 μm film thickness). The oven temperature was set at 50 °C for 2 min and programmed to 200 °C at a rate of 5 °C/min, which was held for 5 min, and then programmed to 330 °C at a rate of 10 °C/min, and finally held for 5 min. The temperatures of the interface and the ion source were set to 280 °C and 250 °C, respectively. Mass spectra were acquired in the mass range of 85–600 *m*/*z* at an ionization voltage of 70 eV.

The resulting data were deconvoluted using the Automated Mass Spectral Deconvolution and Identification System (AMDIS; version 3.2), and the metabolites were identified using the Golm Metabolome Database (GMD) [45]. The data were further processed using SpectConnect with an elution threshold of 1 min and support threshold of 70% [46]. The processed data were normalized using sum normalization and range scaling.

### 2.5. Statistical Analysis and Metabolic Network Analysis

Correlation plot, principal component analysis (PCA), and hierarchical clustering analysis (HCA) were conducted using OriginPro 2023 (OriginLab Corporation, Northampton, MA, USA), SIMCA (version 14.1, Umetrics, Umea, Sweden), and Metaboanalyst 5.0 (https://www.metaboanalyst.ca/, accessed on 19 October 2022), respectively. Differences in metabolites between the groups were evaluated using one-way analysis of variance (ANOVA) followed by Fisher’s LSD post hoc test (*p* < 0.05).

## 3. Results

### 3.1. Polyphenol Content

The content of polyphenols (represented by TP, TET, TFlav, Chl a, Chl b, TAN, and TAC) were significantly different in maple leaves of different colors (Table 1). In this study, the TP and TFlav content were much higher in the red leaves than those in the leaves of the other two colors (green and yellow). The amount of TP in red leaves (106.43 ± 3.828 mg GAE/g) was approximately two times higher than that in the green leaves (49.657 ± 2.501 mg GAE/g), and the TP content of yellow leaves was 69.05 ± 3.59 mg QE/g. The TFlav content of the red leaves (47.48 ± 1.109 mg QE/g) was approximately three times higher than that of the green leaves (17.50 ± 0.87 mg QE/g), and the TFlav content of yellow leaves was 29.60 ± 1.00 mg QE/g. In addition, red leaves contained the highest amount of TET (57.97 ± 3.59 mg GAE/g). There was no significant difference in TET content between the red and yellow leaves (57.41 ± 3.65 mg GAE/g), and it was significantly higher than that of green leaves (39.96 ± 2.65 mg GAE/g). Unsurprisingly, green leaves had more chlorophyll a and b than yellow and red leaves, which is consistent with previous reports [47,48]. In addition, TAN and TAC contents in red leaves were higher than those in the leaves of the other two colors. These results indicate that red maple is a potential source of phenolic compounds.

In the previous study, the similar methods have been widely used to determine the content of TP, TET and TFlav in various plants including maple, *Dendropanax morbifera*, and *Mentha piperita* L. [18,49,50]. The method for measurement of chlorophyll in this study have been used in various plants such as stevia and *Lippia filifolia* [51,52]. The handheld SPAD-502 chlorophyll meter could be used for determining relatively content of chlorophyll and showed the strong correlation with the method used in this study [53]. The pH differential method has been generally used for measurement of TAN content in various plants such as black carrot [54] and the average extension coefficient of carotenoids has been commonly used as the determination of total carotenoid content [55]. Although the methods used in this study have been generally used for measuring the content of polyphenols, the content of each polyphenols should be verified using other techniques such as high performance liquid chromatography (HPLC) and liquid chromatography-mass spectrometry (LC-MS) in further application.

### 3.2. Antioxidant Activity

#### 3.2.1. TBARS

**Figure 2:**
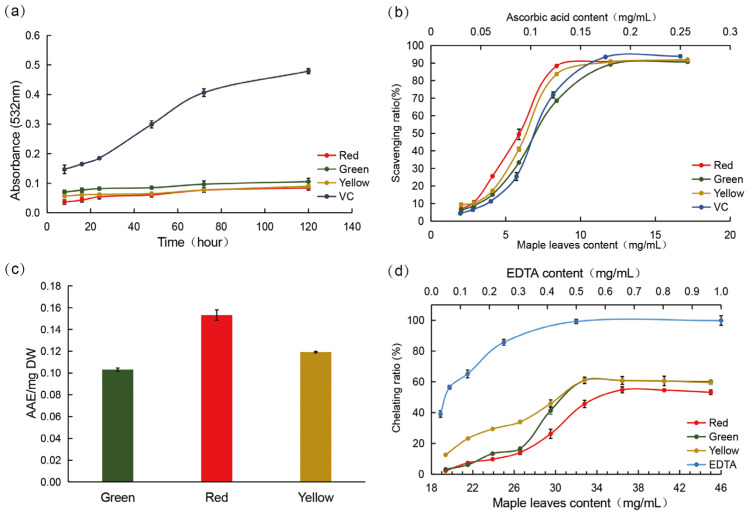
In vitro antioxidant properties of maple leaves of different colors. (**a**) Dose-dependent TBARS absorbance values of maple leaves of different colors; (**b**) Dose-dependent DPPH scavenging ability of maple leaves of different colors and ascorbic acid; (**c**) FRAP value of maple leaves of different colors; (**d**) Dose-dependent metal ions chelation capability of maple leaves of different colors and EDTA. AAE, ascorbic acid equivalents; DW, dry weight.

In this study, we determined the oxidation value of linoleic acid at different time points. As shown in Figure 2a, maple leaves of different colors had reduced lipid oxidation. This indicates that all maple leaves had antioxidant activity, regardless of color. Among these, red leaves showed the highest antioxidant effect. On day 5, the absorbance values (at 532 nm) of all extracts from leaves of different colors were significantly different from those of the control (*p* < 0.01). The absorbance of the red leaves was six times lower than that of the control. In addition, the absorbance value of the red leaves was lower than that of the green leaves (*p* < 0.05).

#### 3.2.2. DPPH-Free Radical Scavenging Effect

The DPPH-free radical scavenging method is currently the most widely used method for determining antioxidant capacity owing to its simplicity, speed, and accuracy. DPPH is one of the most commonly used synthetic free radicals; the higher the IC_50_ value of DPPH, the lower its antioxidant effect. In this study, the DPPH free radical scavenging method was used to determine the free radical scavenging ability of maple leaf extracts [3].

The DPPH scavenging capacities of maple leaves of different colors are shown in Figure 2b. The antioxidant IC_50_ value of the red leaves was 6.53 mg/mL, which was lower than that of the green and yellow leaves. The IC_50_ value of yellow leaves was highest at 7.40 mg /mL, which is equivalent to the antioxidant capacity of ascorbic acid (0.11 mg/mL).

#### 3.2.3. FRAP

The FRAP method is based on the principle that antioxidants reduce Fe^3+^-TPTZ to produce blue-violet Fe^2+^-TPTZ at low pH. Therefore, the absorbance at 593 nm can be used as an indicator of the total antioxidant capacity of the sample [56].

In this study, the FRAP values for maple extracts from the leaves of different colors varied over a wide range of 0.10–0.15 AAE/mg DW (Figure 2c). The FRAP value of red leaves was the highest (0.1532 AAE/mg DW), whereas that of green leaves was the lowest (0.1031 AAE/mg DW). The FRAP value of yellow leaves was 0.119 AAE/mg DW, suggesting that red leaves have a higher antioxidant effect than leaves of the other two colors.

#### 3.2.4. Metal Ions Chelating Ability

Ferrous ions (Fe^2+^) cause lipid peroxidation and food deterioration. Therefore, the ability to chelate ferrous ions can reveal the antioxidant capacity of a substrate [57]. Ferrous ions form a red complex with ferrozine; the intensity of the red color decreases after the addition of chelating agents, which is used as an indicator of the chelating ability and antioxidant capacity of a compound [58].

Figure 2d shows the ferrous ion-chelating ability of maple leaves of different colors. The IC_50_ value of yellow leaves was observed to be the lowest (26.66 ± 1.19 mg/mL), and red leaves showed the weakest ability to chelate ferrous ions (51.02 ± 1.75 mg/mL). This indicates that yellow leaves had a higher antioxidant capacity than the leaves of the other two colors.

The results of the TBARS, DPPH, and FRAP assays demonstrated that red leaves had the highest antioxidant activity among all three colors (red > yellow > green). However, the yellow leaves had the strongest metal-ion chelating ability (yellow > green > red). This finding is similar to the results of previous studies showing that the antioxidant capacity of the same materials may be different based on the methods used [59] owing to the selective reaction of free radicals with antioxidants [60].

### 3.3. Correlation Coefficients of Antioxidant Activities and Polyphenols Content

Previous studies on grapes, *Desmodium* species, and Chinese dark teas [61] have revealed a relationship between polyphenols and antioxidant properties. TBARS, DPPH, and FRAP assays are the most commonly used methods for oxidation resistance testing. The higher the values of TBARS, DPPH, and metal chelation, the weaker the oxidation resistance. Conversely, the higher the value of FRAP, the stronger the oxidation resistance.

**Figure 3:**
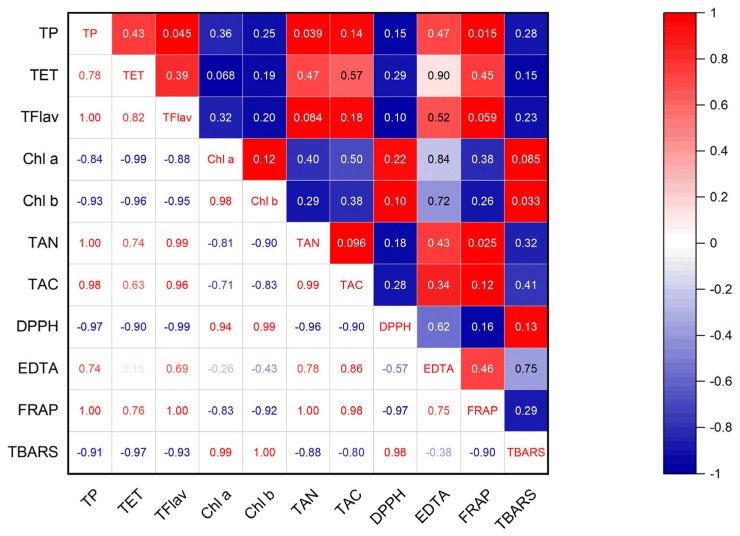
Correlation coefficients of antioxidant activities and polyphenol content. The numbers in the lower triangular represent correlation values; positive values mean positive correlation, whereas negative values mean negative correlation. In the upper triangular, dark red represents significant positive correlation; dark blue means significant negative correlation, and the numbers signify the *p* value (*p* < 0.05).

Figure 3 shows the correlation between the antioxidant activity and polyphenol content. The contents of TP, TET, and TFlav were negatively correlated with TBARS and DPPH values and positively correlated with FRAP values. This indicates that antioxidant activities were highly associated with the content of phenolic compounds and flavonoids in maple leaves. 

Chlorophyll contents (Chl a and Chl b) were positively correlated with TBARS and DPPH values, whereas TAN and TCA were positively correlated with EDTA and FRAP values.

The content of TP, TET, and TFlav in red leaves and its antioxidant capacity (TBARS, DPPH, and FRAP) were higher, whereas the content of chlorophyll (Chl a and b) was lower than that in green and yellow leaves. Collectively, the antioxidant properties were negatively correlated with Chl a and b. On the other hand, they were positively correlated with TP, TET, and TFlav.

### 3.4. Metabolite Profiling

A total of 54 metabolites in the maple leaves of the three colors were identified by GMD. Among these, 23 metabolites were related to antioxidant activity and seven metabolites were polyphenolic acids (Table 2).

**Figure 4:**
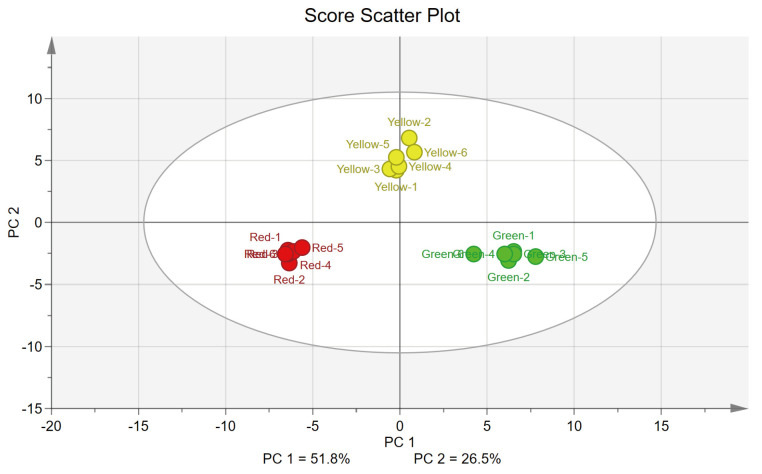
PCA score scatter plot of maple leaves with different colors. Each point on the PCA score scatter plot represents a sample, and each color represents a different color of maple leaves.

To reveal the metabolic differences between maple leaves of different colors, a principal component analysis (PCA) was performed. The PCA score scatter plot (Figure 4) showed a significant difference in the metabolite profiles between the three groups. PC 1 and PC 2 values from the PCA score scatter plot accounted for 78.3% and 69.1% of the total R^2^X (explained) and Q^2^ (predictive) variances, respectively, indicating a good model [84]. As shown in Table 3, the selected 20 metabolites with high absolute loading value which represent how each metabolite contributed to the new variables (PC 1 and PC 2) generated using the PCA model. Among the 53 metabolites identified, 41 metabolites such as succinic acid, inositol, and phenylalanine contributed positively to PC 1 and 26 metabolites contributed positively to PC 2 such as erythritol, γ-tocopherol, and phytol. On the contrary, 12 metabolites including D-fructose, shikimic acid, and D-glucose contributed negatively to PC 1, and 27 metabolites including D-pinitol, trehalose, and fumaric acid contributed negatively to PC 2.

**Figure 5:**
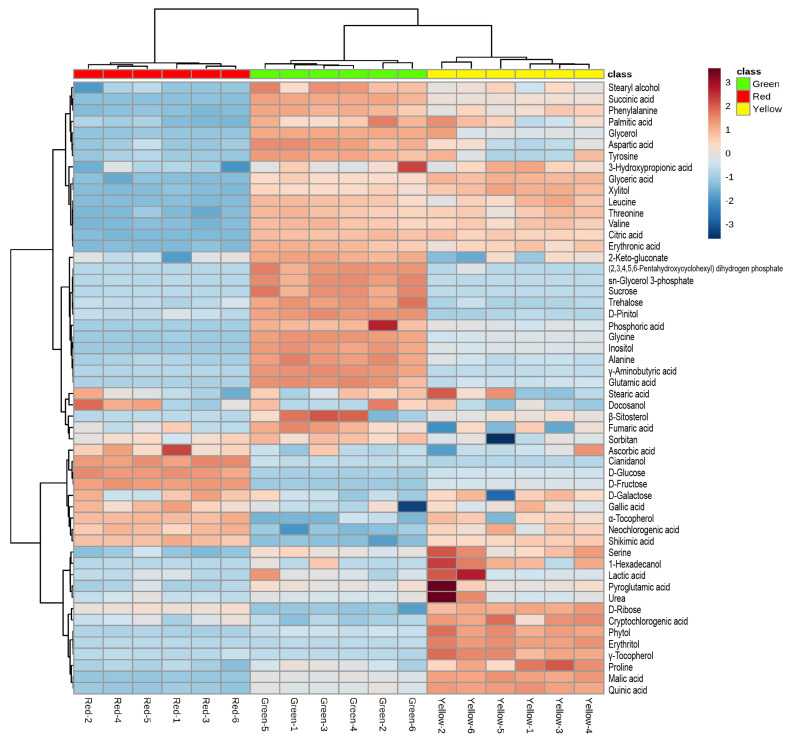
Heatmap from hierarchical clustering analysis (HCA) of maple leaves with different colors.

In the heatmap from HCA, the metabolite profiles of the six biological replicates in each group were similar; however, the profiles varied among the three groups, represented by different colors (Figure 5). The metabolite profiles of maple leaves changed from green to red, and the pattern of yellow leaves was more similar to that of green leaves than of red leaves. Moreover, the relative contents of the different metabolites in each group could be distinguished by the differences in color. One-way ANOVA and Fisher’s LSD post hoc test revealed that the level of 48 metabolites was significantly different between the three groups [*p*-value < 0.05 with false discovery rate (FDR) < 0.05]. Of these 48 metabolites, eight metabolites (including gallic acid, ascorbic acid, and shikimic acid) were higher in red leaves than in green and yellow leaves. Conversely, the levels of 27 metabolites (including threonine, valine, glycine, and fumaric acid) were higher in green leaves than in yellow and red leaves.

By analyzing the metabolites of maple leaves of different colors, we found 48 different metabolites in maple leaves of different colors (*p* < 0.05). The leaves could be divided into three categories by PCA and HCA, indicating that there were significant differences in the metabolite profiles between leaves of different colors.

## 4. Discussion

Phenolic compounds with good antioxidant properties are important parameters for evaluating the antioxidant properties of plant extracts [85]. According to a previous study, the content of polyphenols in various plant leaves ranged from 11.14 to 175.35 mg/g [86]. Among the twenty four plants studied, only seven plants (including *Malus domestica* and *Cydonia oblonga*) had higher polyphenol content than red maple leaves. In this study, the antioxidant activity was measured using four different methods, and we found that the antioxidant capacity of red leaves was much higher than that of the leaves of the other two colors (red > yellow > green). The DPPH scavenging IC_50_ value of red leaves was 6.53 mg/mL. Herbal and low-cost biological resources have been the focus of antioxidant research. In previous research, the DPPH scavenging IC_50_ value of *Cassia fistula* L. seed extract [87], *Gracilaria changii* crude extract [88], and *Erechtites hieraciifolius* [89] was 11.07 mg/mL, 14.70 mg/mL, and 8.46 mg/mL, respectively. In addition, that of various vegetables such as *Murraya koenigii*, *Trigonella foenum-graecum*, *Centella asiatica*, and *Amaranthus* spp. was 9.62 mg/mL, 27.69 mg/mL, 19.89 mg/mL, and 27.27 mg/mL, respectively [90]. Another study showed that the IC_50_ values of various fruits (such as mangosteen, orange, pomelo, grapes, papaya, grape, rose apple, and jackfruit) ranged from 11.18 to 110.46 mg/mL [91]. Compared with the antioxidant properties of various biological materials, the antioxidant properties of maple leaves are not only stronger than those of low-cost biological resources but also stronger than those of some fruits. Therefore, our findings suggest that maple leaves, particularly red leaves, are a good source of polyphenols and antioxidants.

Changes in leaves and carotenoids, among which the degradation of chlorophyll, is a sign of senescence [92,93]. In our observations (as shown in Figure 1), the outer leaves of maple were more converted to red sooner than the inner leaves. This may be because the outer leaves are exposed to more light and higher temperatures during the day and bear lower temperatures at night. This is consistent with previous studies showing that shading slows down the loss of chlorophyll, and the difference in temperature between day and night is conducive to the accumulation of anthocyanins [94,95]. The degradation of chlorophyll, which plays an essential role in capturing light energy, is a critical step in the accumulation of ROS during senescence. Additionally, the synthesis of anthocyanin (which acts as an antioxidant) is an important step in reducing oxidative damage [96,97]. In this study, we compared the chlorophyll content between green, yellow, and red maple leaves. The results agreed with previous reports and demonstrated that the chlorophyll content showed a decreasing tendency from green to red. In contrast, the anthocyanin and carotenoid contents were highest in the red maple leaves. These results are in agreement with a previous study that showed anthocyanin accumulation in red leaves [47]. Previous studies have reported that flavonoid/anthocyanin accumulation is affected by carotenoid accumulation [98] and chlorophyll degradation [99]. Our results also showed a positive and negative correlation between anthocyanins, and carotenoid or chlorophyll content, respectively.

Phenolic compounds are important secondary metabolites in plants that mainly originate from the phenylpropane metabolic pathway [100]. In plants, photosynthetic products generate phosphoenolpyruvic acid (PEP) and erythrose 4-phosphate (E4P) through the Embden-Meyerhof-Parnas (EMP) and pentose phosphate (PPP) pathways, respectively. PEP and E4P enter the shikimic acid pathway and generate phenylalanine, the starting substrate of the phenylphenyne pathway. After a series of enzymatic reactions, flavonoids (such as catechins, proanthocyanidins, and anthocyanins) can be synthesized [100,101]. In this study, we found that the content of phenylalanine in green leaves is the highest, and that the antioxidant-related substances (such as cryptochlorogenic acid, gallic acid, neochlorogenic acid, cianidanol, α-tocopherol, and ascorbic acid) gradually increased during leaf senescence, by further metabolite analysis. These results are consistent with previous results in this study, correlating with higher total phenolic content and stronger antioxidant capacity in red leaves.

In addition, previous studies have reported that the total amount of phenolic acids and antioxidant capacity were low in samples with a high content of free amino acids. The late-harvested sweet potato leaves showed higher antioxidant capacity and polyphenol content, but lower amino acid content than the early- and middle-harvests [102]. Similarly, it was found that the total flavonoid content of *Ocimum basilicum* L. significantly decreased under high amino acid treatment [103]. Consistent with previous studies, our study also showed that most amino acids (such as alanine, aspartic acid, leucine, phenylalanine, threonine, tyrosine, valine, glutamate, glycine, and γ-aminobutyric acid) in green leaves were present at higher levels than those in other colors. However, the polyphenolic acid content and antioxidant capacity were lower, indicating that amino acids may be converted to other phenolic compounds via the shikimic acid/phenylpropanoid/flavonoid synthetic pathway.

## 5. Conclusions

Maples are widely planted and are easily obtained in the northern temperate zone. In this study, we compared the polyphenol content and antioxidant properties of maple leaves of three colors (green, yellow, and red). The levels of TP, TET, TFlav, TAN, and TAC were higher in red leaves than in the other leaves. However, Chl a and b levels were lower in the red leaves. In addition, the antioxidant capacity of the red leaves was higher than that of the green and yellow leaves. PCA and HCA results revealed significant differences in metabolite profiles in maple leaves among green, yellow, and red colors. This study is the first to evaluate the level of polyphenols, antioxidant effects, and metabolites in maple leaves according to color. Therefore, red maple leaves may be used as a potential source of natural antioxidants and could be applied in the development of functional foods and pharmaceuticals.

## Figures and Tables

**Table 1:** Total content of different polyphenols.

	TP (mg GAE/g)	TET (mg GAE/g)	TFlav (mg QE/g)	Chl a (mg/g)	Chl b (mg/g)	TAN (mg/100 g)	TAC (mg/100 g)
**Green**	49.66 ± 2.50 ^a^	39.96 ± 2.65 ^b^	17.50 ± 0.87 ^c^	1.49 ± 0.112 ^a^	0.82 ± 0.12 ^a^	N.D.	0.21 ± 0.01 ^c^
**Yellow**	69.05 ± 3.59 ^b^	57.41 ± 3.65 ^a^	29.60 ± 1.00 ^b^	0.24 ± 0.01 ^b^	0.33 ± 0.02 ^b^	0.02 ± 0.002 ^b^	0.28 ± 0.01 ^b^
**Red**	106.43 ± 3.83 ^c^	57.97 ± 3.59 ^a^	47.48 ± 1.11 ^a^	0.03 ± 0.01 ^c^	0.10 ± 0.02 ^c^	0.07 ± 0.002 ^a^	0.71 ± 0.01 ^a^

Data are expressed as the mean of triplicate analyses ± standard deviation, with different superscripts indicating significant differences between the means (*p* < 0.05). TP, total phenol; TET, total extractable tannin; TFlav, total flavonoid; Chl a, chlorophyll a; Chl b, chlorophyll b; TAN, total anthocyanins; TAC, total carotenoids; N.D., not detected.

**Table 2:** List of identified metabolites.

Scheme	Compound Name	Molecular Formula	*p* Value	FDR	Maximum Value	Minimum Value	Antioxidant & References
Sugars and sugar alcohols (10)	D-Glucose	C_6_H_12_O_6_	<0.05	<0.05	Red	Green	
	D-Fructose	C_6_H_12_O_6_	<0.05	<0.05	Red	Green	
	Sucrose	C_12_H_22_O_11_	<0.05	<0.05	Green	Red & Yellow	
	Trehalose	C_12_H_22_O_11_	<0.05	<0.05	Green	Yellow	△[62]
	D-Galactose	C_6_H_12_O_6_	0.2520	0.2617	Red	Yellow	
	D-Ribose	C_5_H_10_O_5_	<0.05	<0.05	Yellow	Green	
	Inositol	C_6_H_12_O_6_	<0.05	<0.05	Green	Red	
	Erythritol	C_4_H_10_O_4_	<0.05	<0.05	Yellow	Green & Red	▲[63]
	Sorbitan	C_6_H_12_O_5_	<0.05	<0.05	Green	Yellow	
	Xylitol	C_5_H_12_O_5_	<0.05	<0.05	Yellow	Red	△[64]
alcohol, Fatty alcohol and phytosterols (8)	1-Hexadecanol	C_16_H_34_O	<0.05	<0.05	Yellow	Red	▲[65]
	Docosanol	C_22_H_46_O	0.3003	0.3060	Red	Yellow	▲[66]
	Glycerol	C_3_H_8_O_3_	<0.05	<0.05	Green	Red	
	Stearyl alcohol	C_18_H_38_O	<0.05	<0.05	Green	Red	
	D-Pinitol	C_7_H_14_O_6_	<0.05	<0.05	Green	Yellow	▲[67]
	Phytol	C_20_H_40_O	<0.05	<0.05	Yellow	Red	▲[68]
	(2,3,4,5,6-Pentahydroxycyclohexyl) dihydrogen phosphate	C_6_H_13_O_9_P	<0.05	<0.05	Green	Red	
	β-Sitosterol	C_29_H_50_O	0.0655	0.0722	Green	Red	▲[69]
Amino acid (13)	Alanine	C_3_H_7_NO_2_	<0.05	<0.05	Green	Red	
	Aspartic acid	C_4_H_7_NO_4_	<0.05	<0.05	Green	Red	
	Leucine	C_6_H_13_NO_2_	<0.05	<0.05	Green	Red	
	Phenylalanine	C_9_H_11_NO_2_	<0.05	<0.05	Green	Red	
	Proline	C_5_H_9_NO_2_	<0.05	<0.05	Yellow	Red	▲[70]
	Pyroglutamic Acid	C_5_H_7_NO_3_	<0.05	<0.05	Yellow	Red	
	Serine	C_3_H_7_NO_3_	<0.05	<0.05	Yellow	Red	
	Threonine	C_4_H_9_NO_3_	<0.05	<0.05	Green	Red	
	Tyrosine	C_9_H_11_NO_3_	<0.05	<0.05	Green	Red	
	Valine	C_5_H_11_NO_2_	<0.05	<0.05	Green	Red	
	L-Glutamate	C_5_H_9_NO_4_	<0.05	<0.05	Green	Red	△[71]
	Glycine	C_2_H_5_NO_2_	<0.05	<0.05	Green	Red	
	γ-Aminobutyric acid	C_4_H_9_NO_2_	<0.05	<0.05	Green	Red	▲[72]
Organic acid (14)	Malic acid	C_4_H_6_O_5_	<0.05	<0.05	Yellow	Red	
	2-Keto-gluconate	C_6_H_10_O_7_	<0.05	<0.05	Green	Yellow	
	3-Hydroxypropionic acid	C_3_H_6_O_3_	<0.05	<0.05	Green	Yellow	
	Lactic acid	C_3_H_6_O_3_	0.1433	0.1517	Yellow	Red	
	Palmitic acid	C_16_H_32_O_2_	<0.05	<0.05	Green	Red	
	Quinic acid	C_7_H_12_O_6_	<0.05	<0.05	Yellow	Red	△[73]
	sn-Glycerol 3-phosphate	C_3_H_9_O_6_P	<0.05	<0.05	Green	Red	
	Stearic acid	C_18_H_36_O_2_	0.58722	0.58722	Green	Yellow	△[74]
	Succinic acid	C_4_H_6_O_4_	<0.05	<0.05	Green	Red	
	Ascorbic acid	C_6_H_8_O_6_	<0.05	<0.05	Red	Yellow	▲[75]
	Citric acid	C_6_H_8_O_7_	<0.05	<0.05	Green	Red	△[76]
	Glyceric acid	C_3_H_6_O_4_	<0.05	<0.05	Yellow	Red	
	Erythronic acid	C_4_H_8_O_5_	<0.05	<0.05	Green	Red	
	Fumaric acid	C_4_H_4_O_4_	<0.05	<0.05	Green	Yellow	△[77]
Polyphenols and Phenolic acids (7)	Cryptochlorogenic acid	C_16_H_18_O_9_	<0.05	<0.05	Yellow	Green	▲[78]
	Gallic acid	C_7_H_6_O_5_	<0.05	<0.05	Red	Green	▲[79]
	Neochlorogenic acid	C_16_H_18_O_9_	<0.05	<0.05	Red	Green	▲[78]
	Cianidanol	C_15_H_14_O_6_	<0.05	<0.05	Red	Yellow	▲[80]
	α-Tocopherol	C_29_H_50_O_2_	<0.05	<0.05	Red	Green	▲[81]
	γ-Tocopherol	C_28_H_48_O_2_	<0.05	<0.05	Yellow	Green	▲[82]
	Shikimic acid	C_7_H_10_O_5_	<0.05	<0.05	Red	Green	▲[83]
Others (2)	Phosphoric acid	H_3_PO_4_	<0.05	<0.05	Green	Red	
	Urea	CH_4_N_2_O	0.1101	0.1189	Yellow	Red	

Note: FDR: False Discovery Rate. △: metabolites related to antioxidant activity. ▲: metabolites with antioxidant activity.

**Table 3:** List of twenty metabolites with high absolute loading values on PC 1 and PC 2.

PC 1		PC 2	
Metabolites	Loadings Scores	Metabolites	Loadings Scores
Succinic acid	0.188	Erythritol	0.262
Inositol	0.184	γ-Tocopherol	0.26
Phenylalanine	0.183	Phytol	0.259
Glycerol	0.182	Quinic acid	0.231
Glycine	0.181	Malic acid	0.229
D-Fructose	−0.179	1-Hexadecanol	0.209
Erythronic acid	0.179	Proline	0.202
Aspartic acid	0.178	Serine	0.195
Shikimic acid	−0.178	Cryptochlorogenic acid	0.195
Citric acid	0.177	D-Pinitol	−0.188
Valine	0.176	Trehalose	−0.187
Glutamic acid	0.174	D-Ribose	0.184
D-Glucose	−0.174	Fumaric acid	−0.175
Threonine	0.173	Sorbitan	−0.174
Neochlorogenic acid	−0.173	2-Keto-gluconate	−0.163
Alanine	0.172	Urea	0.159
Tyrosine	0.171	Glyceric acid	0.158
Leucine	0.168	Xylitol	0.156
Stearyl alcohol	0.168	Pyroglutamic Acid	0.156
Phosphoric acid	0.167	Cianidanol	−0.144

Note: Loadings scores were obtained by PCA analysis, presenting data as the 20 with the largest absolute values in the loading values, and data analysis was analyzed in Metaboanalyst.

## Data Availability

Data is contained within the article.
